# Chicken γδ T cells proliferate upon IL-2 and IL-12 treatment and show a restricted receptor repertoire in cell culture

**DOI:** 10.3389/fimmu.2024.1325024

**Published:** 2024-02-13

**Authors:** Antonia E. Linti, Thomas W. Göbel, Simon P. Früh

**Affiliations:** ^1^ Department of Veterinary Immunology, Ludwig-Maximilians-Universität München, Munich, Germany; ^2^ Department of Veterinary Medicine, Institute of Virology, Freie Universität Berlin, Berlin, Germany

**Keywords:** chicken γδ TCR, IL-2, IL-12, *in vitro* culture, γδ T cell subsets, TCR repertoire analysis

## Abstract

In chickens, γδ T cells represent a large fraction of peripheral T cells; however, their function remains largely unknown. Here, we describe the selective *in vitro* expansion of γδ T cells from total splenocytes by stimulation with the cytokines IL-2 and IL-12. Under these conditions, γδ T cells proliferated preferentially and reached frequencies of >95% within three weeks. Although IL-2 alone also triggered proliferation, an increased proliferation rate was observed in combination with IL-12. Most of the expanded cells were γδ TCR and CD8 double-positive. Splenocytes sorted into TCR1^+^CD8^+^, TCR1^high^CD8^−^, and TCR1^low^CD8^−^ subsets proliferated well upon dual stimulation with IL-2/IL-12, indicating that none of the three γδ T cell subsets require bystander activation for proliferation. TCR1^+^CD8^+^ cells maintained CD8 surface expression during stimulation, whereas CD8^−^ subpopulations showed varied levels of CD8 upregulation, with the highest upregulation observed in the TCR1^high^ subset. Changes in the γδ T-cell receptor repertoire during cell culture from day 0 to day 21 were analyzed by next-generation sequencing of the γδ variable regions. Overall, long-term culture led to a restricted γ and δ chain repertoire, characterized by a reduced number of unique variable region clonotypes, and specific V genes were enriched at day 21. On day 0, the δ chain repertoire was highly diverse, and the predominant clonotypes differed between animals, while the most frequent γ-chain clonotypes were shared between animals. However, on day 21, the most frequent clonotypes in both the γ and δ chain repertoires were different between animals, indicating that selective expansion of dominant clonotypes during stimulation seems to be an individual outcome. In conclusion, IL-2 and IL-12 were sufficient to stimulate the *in vitro* outgrowth of γδ T cells. Analyses of the TCR repertoire indicate that the culture leads to an expansion of individual T cell clones, which may reflect previous *in vivo* activation. This system will be instrumental in studying γδ T cell function.

## 1 Introduction

In birds and mammals, T lymphocytes play a pivotal role in the adaptive immune system. The two distinct groups, αβ and γδ T cells, express different T cell receptors (TCRs) on their surfaces, which are heterodimers composed of either an alpha and a beta chain, or a gamma and a delta chain, respectively (1–3). Each chain consists of a constant region and a variable region, which are uniquely created during thymic maturation of T cells by somatic DNA recombination of germline V(D)J genes.

In chickens, the genes for TCRγ can be found on chromosome 2, for TCRβ on chromosome 1, and for TCRα and TCRδ on chromosome 27 (4–9), where the δ genes are nested between the TCRα genes (10). In chickens and mammals, some variable (V) α gene segments can be joined with diversity (D) and joining (J) genes of the δ locus, which further increases the combinatorial diversity (4, 10–12). Additionally, a second TCRδ locus on chromosome 10 with a single set of V, D, J, and constant (C) genes has been described in chickens (13).

Chicken T cells can be identified at the protein level by monoclonal antibody clones TCR1, TCR2, and TCR3, which bind γδ, Vβ1 αβ, and Vβ2 αβ T cells, respectively (14–16).

The functions and effector mechanisms of αβ T cells have been well-characterized in many species. Most αβ T cells recognize peptides presented by MHCI or MHCII molecules on the surface of antigen-presenting cells. There are two main subsets: CD4^+^ T helper αβ T cells that recognize peptides presented by MHCII and CD8^+^ cytotoxic αβ T cells that recognize peptides presented by MHCI (17, 18). On the other hand, γδ T cells are not restricted to peptides presented by MHCI or MHCII molecules; instead, they can also directly recognize soluble or unprocessed antigens and nonpeptide antigens, such as glycoproteins. Human Vγ9Vδ2^+^ T cells are activated by phosphoantigens (19–21). In addition, γδ T cells recognize stress-induced ligands that are upregulated in cells in response to infection and stress, leading to different effector functions such as the orchestration of pathogen clearance (17, 22). *In vitro* studies have further shown that some γδ T cells have cytotoxic effector functions (23) and that they can also present antigens to B cells (24).

γδ T cells with innate cell-like features frequently participate in innate immune responses (25–28) and play an important role in tumor surveillance, tissue healing, and protection against intra- and extracellular pathogens (17). Human, mouse, and rat are the so-called γδ low species that are characterized by a γδ T cell frequency of 1%–10% of all T lymphocytes in peripheral blood (29–31). Pigs (32), cattle, sheep (33, 34), goats (35), and chickens, on the other hand, are considered γδ high species. Up to 50% of all circulating T lymphocytes in chicken blood are γδ T cells (16), and a large γδ T cell population is found in different tissues, including the intestine and spleen, particularly in the red pulp (36, 37). γδ T cells are the first T cells generated in the chicken thymus during embryonic development (16).

In chickens, γδ T cell frequencies are influenced by the sex and age of the animals. Male chickens, for example, show androgen-induced expansion of γδ T cells in the peripheral blood and spleen between 4 months and 6 months of age (38). Despite the overall high frequency of γδ T cells in chickens, little is known about their functions. Previous studies have shown that γδ T cells can produce a range of cytokines and interferons, such as IL-10 and IFN-γ (39), and exert cytotoxic effector functions (40). γδ T cells of MDV-vaccinated chickens exhibit high cytotoxic activities *ex vivo* (41), and chicks infected with *Salmonella typhimurium*, for example, show an expansion of CD8αα positive γδ T cell subsets in the blood and different organs (42, 43).

A high percentage of chicken γδ T cells express CD8 on their surfaces in the spleen but only a small percentage in the blood (14, 37). In addition to functioning as a coreceptor for TCR antigen recognition (44), CD8 supports T cell activation through interaction with an intracellular tyrosine protein kinase (45, 46). Previous studies have shown that splenic chicken γδ T cells can be stimulated by either IL-2 and Concanavalin A (47) or by a combination of receptor ligation and cytokine-containing tissue culture supernatants when cultured together with αβ T cells (37). The responding γδ T cells express CD8 on their surface. IL-2 mainly promotes the proliferation of CD8^+^ cells (48). In mice, CD8 expression in γδ T cells seems to occur due to activation with IL-2 and Concanavalin A only in the presence of αβ T cells (49).

Investigating the repertoire of gamma and delta TCRs in chickens and their behavior in cell culture will contribute to a better understanding of this important cell type. In-depth characterization of (clonal) γδ T cell populations requires TCR repertoire analyses for both γ and δ chains, which has previously not been possible due to an incomplete annotation of the δ locus in the chicken genome.

Previous repertoire analyses of γ chains in chickens revealed that the TCRγ repertoire is largely composed of highly public CDR3 sequences formed by a wide range of V segments, with a higher proportion of private sequences in tissues, such as the spleen and thymus (8, 50). On the other hand, γδ T cells often exhibit tissue specificity in their expression of invariant TCRs, for example, Vγ5Vδ1 TCR in the skin of mice (17).

In this paper, we describe long-term IL-2 and IL-12 driven culture of chicken splenic γδ T cells, characterization of cultured cells, and TCR repertoire analyses at different time points during cell culture.

## 2 Materials and methods

### 2.1 Ethics statement

All animal research projects were sanctioned by the Government of Upper Bavaria (identification code: 55.2-1-54-2532.0-60-2015; June 2019). All animal procedures were performed in accordance with the regulations and guidelines established by the Committee and the International Standards for Animal Welfare.

### 2.2 Animals

Fertilized eggs from the chicken line M11 (B²/B²) were obtained either from S. Weigend (Federal Research Institute for Animal Health, Mariensee, Germany) or from our own breeding. They were hatched and maintained under conventional conditions at the Institute for Animal Physiology, LMU Munich (Germany). Animals received food and water *ad libitum*. Experiments were performed at the age of 6 weeks–41 weeks in both female and male animals.

### 2.3 Cytokines

Chicken recombinant IL-2 cytokine was produced as previously described (40). Recombinant chicken IL-12 was produced in a stable IL-12 producing HEK293 cell line. Chicken IL-12p35 and IL-12p40 sequences fused by a glycine–serine linker (40, 51), were cloned into a pcDNA3.1 vector together with an HA-signal peptide and an N-terminal FLAG epitope, and the plasmid was used for the stable transfection of HEK 293 cells using Metafectene^®^ (Biontex). Transfected cells were incubated at 37°C and 5% CO2 for 24 h and selected as stable transfectants with G418 at a concentration of 800 µg/ml. The supernatant of stable transfectants was tested using sandwich ELISA for the presence of FLAG-tagged recombinant chicken IL-12. Finally, IL-12 producing cells were cultured in a bioreactor (Wheaton^®^ Celline™ Bioreactors: Celline 1000 Adherent). The biological activity and optimal dilution for both cytokines were evaluated using bromodesoxyuridin (BrdU) proliferation assay (Cell Proliferation ELISA, BrdU (chemiluminescence); Roche) with chicken splenocytes (freshly isolated and cryopreserved).

### 2.4 Cell preparation and cell culture

Splenocytes were obtained by passing the chicken spleen through a stainless-steel mesh, followed by density gradient centrifugation of the single-cell suspension using Ficoll Histopaque^®^-1077 (Sigma-Aldrich GmbH). Freshly isolated cells were either used directly for RNA isolation and flow cytometry staining or cultivated at a density of 1 × 10^6^ cells per well in flat-bottomed 96 well plates with RPMI cell culture medium containing 8% Fetal Bovine Serum (FBS), 2% chicken serum (ChS), and 1% penicillin/streptomycin (P/S), stimulated with recombinant IL-2 and IL-12, at 40°C and 5% CO2. Cells were treated by demi-depletion every two to three days with fresh cell culture medium and fresh cytokines.

### 2.5 Antibodies for flow cytometry and fluorescence activated cell sorting

Chicken splenocytes were stained with the Fixable Viability Dye eFluor 780 (eBioscience™ Fixable Viability Dye e Fluor™ 780; Invitrogen) to distinguish between live and dead cells. For staining of CD8 positive and γδ TCR positive cells, anti CD8-PE (clone CT-8, Phycoerythrin conjugate, mouse IgG1k) and anti TCR1-FITC (clone TCR-1, fluorescein isothiocyanate conjugate, mouse IgG1k) (16) antibodies were obtained from Southern Biotechnology Associates (SBA). The cells were analyzed using a FACS Canto II instrument, 10.000 Events (single cells) were collected in every experiment. The gating strategy is shown in the **Supplementary Material** (**Supplementary Figure 1**). Fluorescence-activated cell sorting was performed with a FACSAria III instrument (BD) using an 80 μm nozzle. The measurements were analyzed using FlowJo™ v10.8.1 Software (BD Life Sciences) (52). Sort-purified cells were counted using a hemocytometer and cultured at 6 × 10^5^ cells per well with IL-2 and IL-12, and their proliferation was measured using a BrdU proliferation assay.

### 2.6 BrdU proliferation assay

BrdU proliferation assay is a non-radioactive DNA assay for the quantification of cell proliferation. Splenocytes, either freshly isolated, cryopreserved, or sort-purified γδ T cells, were cultivated in black 96-well plates with a clear bottom (ViewPlate™—96 F TC, PerkinElmer) for 72 h with or without IL-2 and IL-12. Next, the cells were labeled with BrdU labeling reagent for another 16 h at 40°C and 5% CO2, during which BrdU was incorporated into the cellular DNA during DNA synthesis in replicating cells. The plates were then dried at 60°C, followed by denaturation and fixation, incubation with anti-BrdU-POD working solution for 90 min, and labeling of the cells with a fluorescent dye. The 96-well plates were measured using a luminometer (Glomax, Promega), and raw measurements in rlu/s (relative light units/s) were analyzed using Excel. The proliferation index (PI) was calculated by dividing the measured fluorescence of stimulated cells by the fluorescence of the unstimulated control cells. Standard deviations were calculated using the Excel software.

### 2.7 RNA isolation, cDNA synthesis, and semi-nested PCRs

To investigate the repertoire of γδ T cells, a next-generation sequencing method for the whole γδ V region was devised (53, 54) (Schematic explanation of the NGS approach is shown in **Supplementary Figure 2**). RNA was extracted from 1 × 10^7^ cells per condition on day 0 and on day 21 (RNeasy^®^ Mini Kit and RNase-Free DNase Set; Qiagen). The quality and quantity of the isolated RNA were measured using a Bioanalyzer 2100 Expert (Agilent) and a NanoDrop ND-1000 (PeqLab). Only RNA samples with RIN values above 9 and 260/280 and 260/230 ratios greater than 1.8 were used for further processing. Reverse transcription and TCR amplicon generation were performed following chicken-specific adaptation of the approach described by Mamedov et al. (53). In brief, between 350 ng and 400 ng of RNA were reverse transcribed to cDNA by 5’RACE with a SMARTScribe Reverse Transcriptase (Takara) using reverse primers specific to the constant C-region of the gamma and the delta chain in one reaction and a Template Switch Oligonucleotide containing a Unique Molecular Identifier (UMI) (**Supplementary Table 1**). All primers used are listed in the **Supplemental Materials** (**Supplementary Figure 2**, **Supplementary Table 2**). The barcoding of cDNA allowed us to filter out PCR duplicates *in silico*, leading to more precise quantitative analysis.

1 μl of γδ cDNA was amplified in two semi-nested PCRs using an Advantage2 Polymerase (Takara) and gene-specific reverse primers (Primers in **Supplementary Table 2**, PCR conditions in **Supplementary Table 3**). Amplification of gamma and delta chains was conducted in one PCR reaction and the PCR products were purified with magnetic beads (Beckman Coulter™ Agencourt AMPure XP Beads) using a ratio of 1:0.65 (DNA: beads) and eluted in 25 μl nuclease-free water. The second PCR was performed in separate reactions for γ and δ. 1μl of purified product from the first PCR was used as a template for the second PCR and the amplicons were separated by electrophoresis on an agarose gel (1% agarose low EEO (Agarose Standard) [Applichem Pancreac] in 1 × TBE buffer) and gel-purified using the Wizard^®^ SV Gel and PCR Clean-Up System (Promega).The purified samples with attached Universal Adapters were sent to Eurofins for paired-end Illumina sequencing with a read length of 2 × 300 bp and a guaranteed output of 60,000 paired-end reads per sample. Data were delivered as fastq-files.

### 2.8 Bioinformatic analysis

The fastq-files were further analyzed with a bioinformatic pipeline using FastQC 0.12.0 (55) for quality control, MiXCR v4.3.2 (56, 57), for the alignment and Immunarch 1.0.0 (58) in R v4.2.2 (59) for the graphical representation. The bioinformatic pipeline and annotation of the Huxu chicken genome (60) for the alpha, beta, gamma, and delta V gene segments were established by S. Früh (manuscript in preparation). The raw data (fastq-files) were uploaded to the SRA database and are accessible via the following accession number: PRJNA1054968.

### 2.9 Statistics

Statistical analyses were performed using R v4.2.2. To compare the differences between the two dependent conditions, paired Student’s t-test was performed (used in **Supplementary Figure 9**). Unpaired Student’s t-test was performed to compare the differences between two independent conditions (used in **Supplementary Figure 10**). To compare differences between more than two dependent conditions, one-way repeated measures ANOVA with Tukey’s HSD test as a *post-hoc* test was performed (used in **Supplementary Figures 3**, **4**). Statistical significance was set at P ≤0.05.

## 3 Results

### 3.1 γδ T cells proliferate *in vitro* after IL-2/IL-12 stimulation of splenocytes

**Figure 1:**
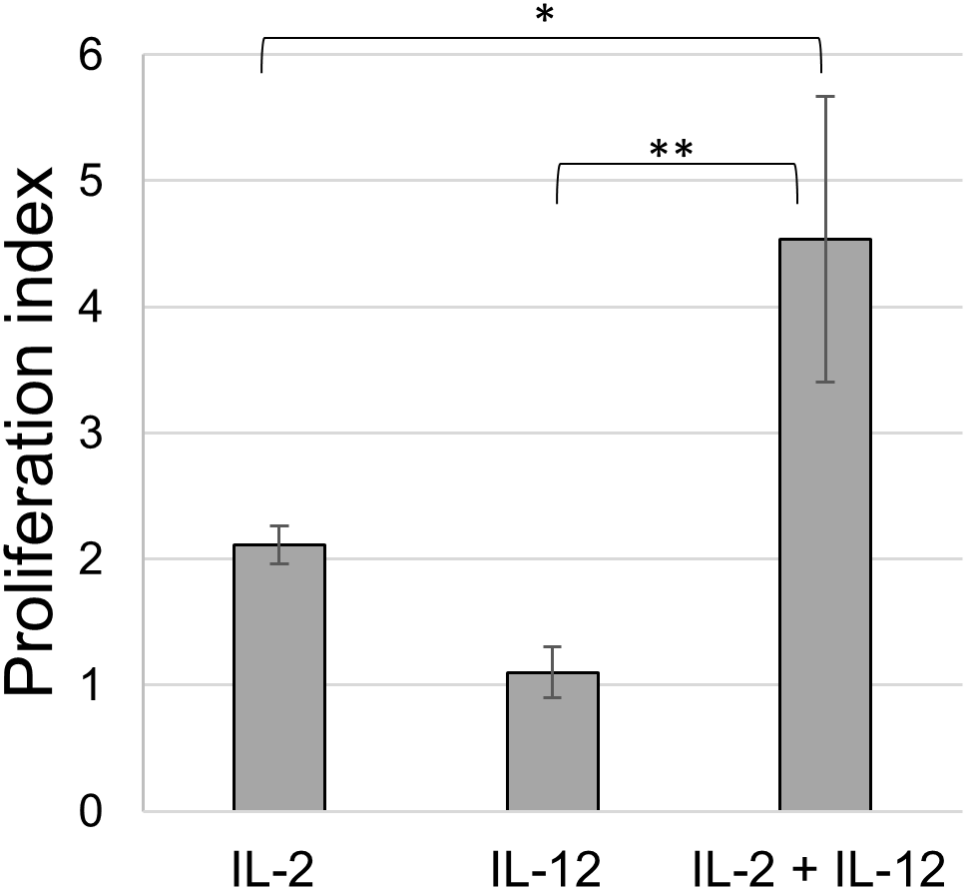
Proliferation of splenocytes upon cytokine stimulation. BrdU assay of splenocytes stimulated with IL-2 alone (1:800), IL-12 alone (1:80) and of a combination of both (IL-2 1:800 and IL-12 1:80); animals = 3. Mean ± SD. p-values as indicated, *p ≤0.05, **p ≤0.01.

Initially, we tested the cytokines IL-2 and IL-12 for their potential to induce the proliferation of freshly isolated splenocytes. Different seeding densities in a flat-bottomed 96-well plate, cell culture media, and concentrations of IL-2 and IL-12 were tested. IL-2 alone induced strong splenocyte proliferation, as measured in a BrdU assay, with the highest proliferation (PI: 2) observed at a dilution of 1:800 (**Supplementary Figure 3A**). In contrast, the proliferation of splenocytes induced by IL-12 alone was lower (PI between 1 and 0.9 at dilutions of 1:10 to 1:160), with the highest proliferation observed at a 1:80 dilution (**Supplementary Figure 3B**). Next, we tested whether a combination of these cytokines could further increase proliferation. IL-2 was used at an optimal dilution of 1:800, and IL-12 was added at various dilutions to determine whether proliferation could be further enhanced (**Supplementary Figure 3C**). In this assay, the proliferation of splenocytes was more than twice as high as that of IL-2 or IL-12 alone, reaching PI values of almost 5, when IL-2 at 1:800 was combined with IL-12 at 1:80 (**Figure 1**). These cytokine concentrations were used throughout the study, employing the same batch of cytokine preparations.

We also compared different cell densities ranging from 2.5 × 10^5^ cells per well and 5 × 10^5^ cells per well to 1 × 10^6^ cells per well, with the best results obtained at 1 × 10^6^ cells per well (**Supplementary Figure 4A**). In addition, three cell culture media (IMDM + 8%FBS + 2%ChS + 1%P/S, RPMI + 8%FBS + 2%ChS + 1%P/S, and RPMI + 10%FBS + 1%P/S) were tested using a BrdU proliferation assay, and the highest proliferation indices were observed in RPMI + 8%FBS + 2%ChS + 1%P/S (**Supplementary Figure 4B**).

**Figure 2:**
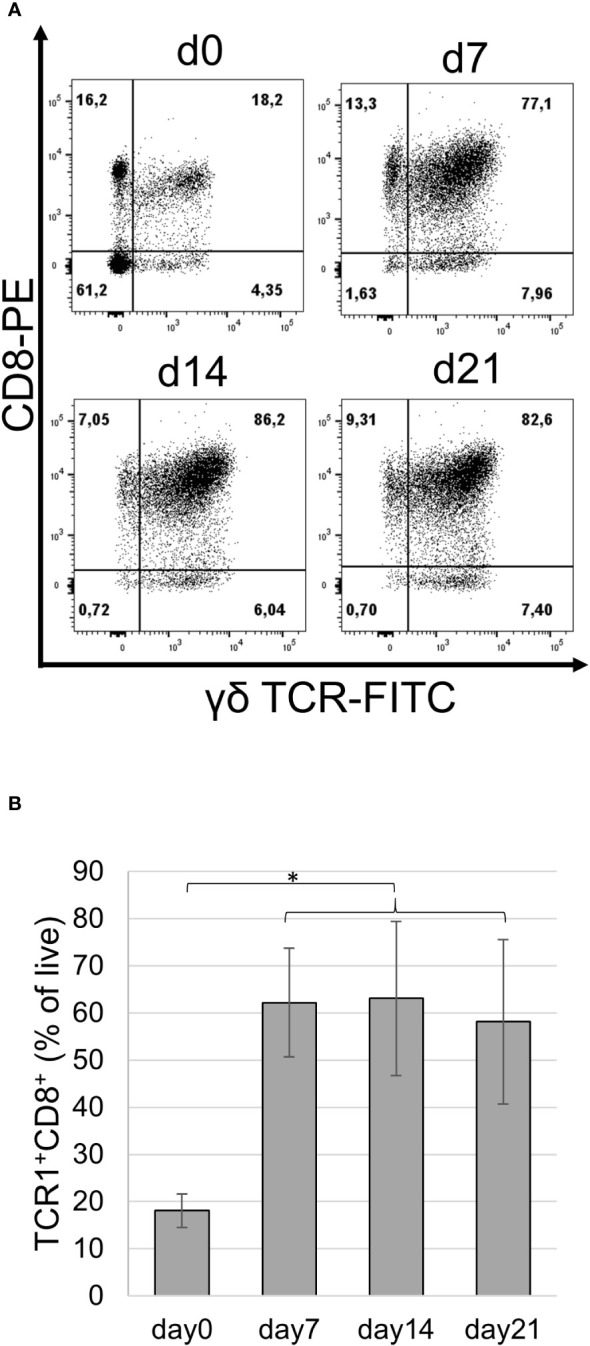
Phenotype of IL-2/IL-12 stimulated splenocytes. Splenocytes were stained with TCR1 and CT8 mAbs before culture and on days 7, 14, and 21 (d0, d7, d14, and d21) following repetitive cytokine stimulation. **(A)** Frequency of cell populations as a percentage of live single cells is indicated. Data from one female chicken representative of three experiments are shown. **(B)** Frequency of TCR1^+^CD8^+^ cells over time in cultured splenocytes from three different animals. Mean ± SD; p-values as indicated, *p ≤0.05.

In the next set of experiments, we attempted to extend the culture time of proliferating cells by propagating the cells at an optimal cell density and feeding with fresh medium containing new cytokines every two to three days. These cultures were maintained for up to 3 weeks, with cell viability slightly decreasing towards day 21 (**Supplementary Figure 5**). Notably, after 21 days of culture, most cells were γδ T cells, reaching frequencies up to 90%. Double staining of cells cultured for different time periods using the TCR1 mAb in combination with anti-CD8 demonstrated that the frequency of double-positive cells significantly increased in all tested animals of different sexes and reached values of up to 86% (**Figures 2A, B**).

These data suggest that the combination of IL-2 and IL-12 in splenocyte cultures favors the selective outgrowth of γδ T cells that predominantly express CD8.

### 3.2 Different γδ T cell populations respond to cytokine stimulation

**Figure 3:**
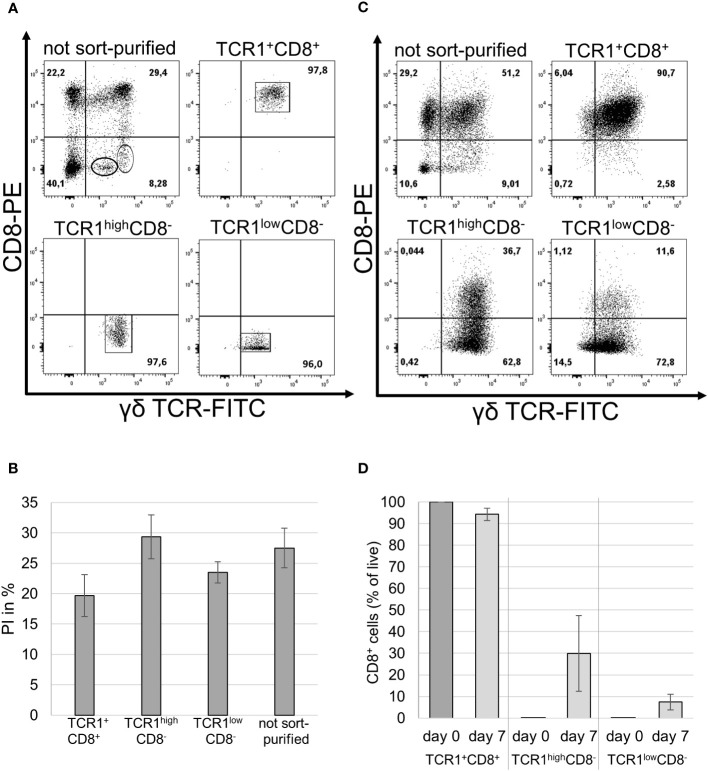
Phenotype of IL-2/IL-12 stimulated sort-purified γδ T cell populations. **(A)** Purified splenocytes were stained prior to sorting using TCR1 and CT8 mAbs (top left panel) and the purity of the three sorted γδ T cell populations after sort-purification (other panels). One representative experiment is shown, with the frequencies indicated. n = 3 biological replicates. **(B)** The BrdU assay of sort-purified and unpurified cells stimulated with IL-2/-12. PI was expressed as the percentage of total proliferation. Mean ± SD; n = 3 biological replicates; p-values after statistical analysis of the rlu/s of the stimulated sorted and unsorted populations showed no significant differences in their proliferation capacities (p >0.05 = ns). **(C)** Frequency of IL-2/IL-12 stimulated sort-purified and unpurified populations stained after 7 days in cell culture using TCR1 and CT8 mAbs. One representative of three experiments is shown. **(D)** Frequency of TCR1^+^CD8^+^ cells in the three sorted subpopulations on day 0 and after 7 days in cell culture with IL-2 and IL-12. n = 3 male animals. Mean ± SD.

In previous experiments, splenocytes were used for *in vitro* culture. Thus, it cannot be excluded that the cytokines, rather than directly affecting γδ T cells, had an indirect effect on bystander cells. Therefore, the splenocytes of the three male animals were sorted according to their TCR1/CD8 profiles into three distinct subsets. CD8^−^ γδ T cells can be further divided into TCR1^low^ and TCR1^high^ subsets. In addition, the TCR1^+^CD8^+^ subset was sorted, and these three populations, together with unsorted cells, were subsequently cultivated with IL-2 and IL-12 (**Figure 3A**). The purity of the sort-purified cells was higher than 95% for all populations (**Figure 3A**).

A BrdU proliferation assay was performed on different sort-purified populations and unsorted cells as a control (**Figure 3B**). Cells from each population were divided into two groups: one group was stimulated with IL-2 and IL-12 and the other was left unstimulated as a negative control. The negative controls proliferated less effectively than the stimulated cells and exhibited low rlu/s. For the three sort-purified populations and unsorted control cells, the assay showed equally high proliferation indices in the animals tested (**Figure 3B**). Stimulated cells proliferated better than unstimulated controls, while the proliferation capacities of sorted and unsorted stimulated cells were not significantly different.

The cells were reanalyzed by staining and flow cytometry using TCR1 and CT8 mAbs after one week of stimulation with IL-2 and IL-12. Unsorted cells were predominantly TCR1/CD8 double-positive and sorted TCR1^+^CD8^+^ cells retained their double-positive phenotype (**Figure 3C**). Interestingly, on average, about one-third of the TCR1^high^CD8^−^ cells started to express the CD8 antigen, whereas only a small fraction of the TCR1^low^CD8^-^ cells expressed CD8 after stimulation (**Figures 3C, D**).

In conclusion, IL-2 and IL-12 appear to have a direct effect on γδ T cell proliferation. Different γδ T cell subsets based on TCR γδ and CD8 expression equally react to stimulation with cell proliferation without bystander activation. CD8^-^ subsets start to express CD8 upon cytokine stimulation to varying degrees in the three animals.

### 3.3 Cytokine stimulation of γδ T cells leads to a restricted TCR repertoire

As a next step, we wanted to characterize the γδ TCR repertoire of IL-2/IL-12 stimulated total splenocytes to determine whether specific subsets were preferentially responding. Thus, we analyzed the TCR repertoire before stimulation and after three weeks of cell culture. We used 5’RACE and PCRs to selectively amplify the expressed TCRγδ repertoire of three animals of different sexes at days 0 and 21 of cell culture. Amplicons were sequenced on the Illumina platform and the sequences obtained were annotated by alignment to the chicken germline V(D)J genes using MiXCR v4.3.2. Using this approach, more than 90% of the gamma chain sequences and 80% of the delta chain sequences aligned successfully to the reference (**Supplementary Figure 6**).

**Figure 4:**
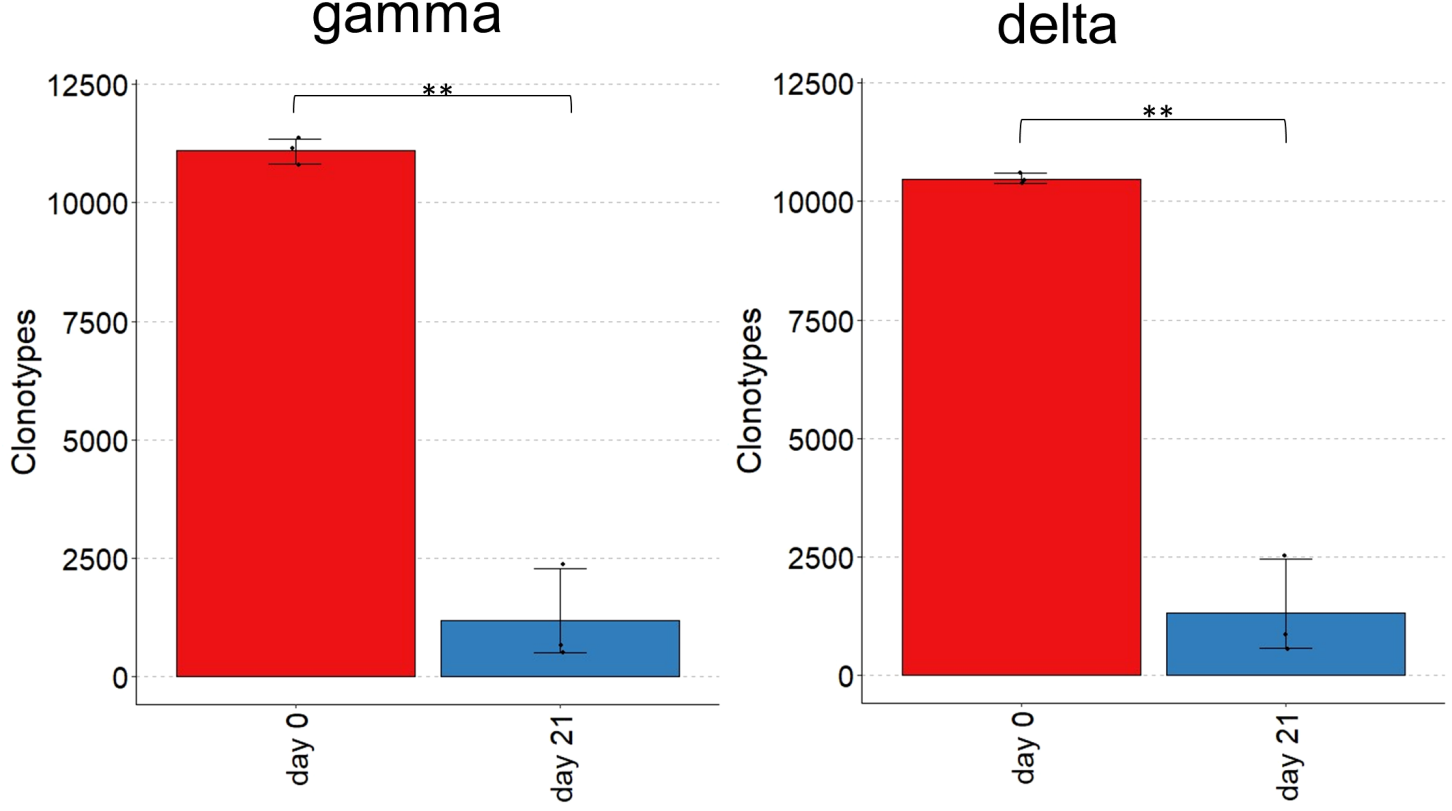
Number of unique clonotypes in TCR γ and δ repertoires of cultured splenocytes. Barplot showing the number of unique clonotypes before and after IL-2/IL-12 stimulation of splenocytes on days 0 and 21. n = 3 biological replicates; p-values as indicated, ** = p ≤0.01.

Gamma and delta chain repertoires changed drastically during stimulation over the course of 21 days. Overall, long-term culture led to fewer expressed V-regions (**Figure 4**). Approximately 11,000 unique γ-chain clonotypes were expressed on day 0, which reduced to 1,200 on day 21. Of the approximately 10,000 unique δ chain clonotypes on day 0, only 1,300 remained on day 21. The numbers of gamma and delta chain clonotypes were correlated, as the animals with a more restricted gamma repertoire also had a more restricted delta repertoire and vice versa (**Supplementary Figure 7**).

**Figure 5:**
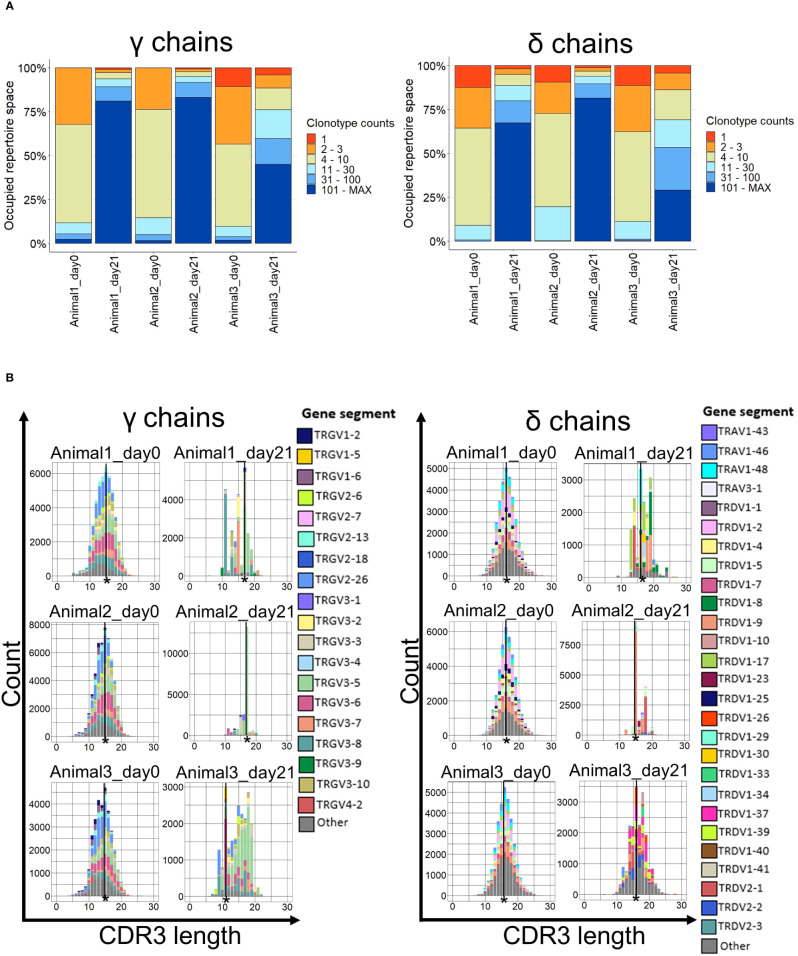
Clonality and CDR3 spectratypes of TCR γ and δ repertoires. **(A)** The occupied repertoire space of clonotype groups based on the total count of each unique clonotype in percent. n = 3 biological replicates; **(B)** CDR3 spectratypes (Histograms of the CDR3 lengths in amino acids with the number of occurrences on the y-axis) of the γ and δ chains on days 0 and 21. n = 3 biological replicates. The most common CDR3 length is indicated (*).

Most of the V regions expressed on day 21 appeared at a higher frequency than those on day 0 (**Figure 5A**). This was clearly recognizable in animals 1 and 2, where more than 75% of the γ repertoire was occupied by clonotypes with more than 100 counts (**Figure 5A**). The same trend, albeit less pronounced, was also observed in the third animal and delta-chain clonotypes (**Figure 5A**).

The CDR3 clonotype distribution plotted by CDR3 length (spectratype) was approximately normally distributed at day 0 but strongly skewed on day 21 for both γ and δ chains (**Figure 5B**). The most prevalent CDR3 length on day 0 was 15 amino acids (aa) for γ chains and 16 aa for δ chains in all animals. After stimulation, the most common γ chain CDR3 length was 17 aa in two animals and 11 aa in the third, whereas for delta, the CDR3 length either did not change (two animals) or shifted to 15 aa in the third animal (**Figure 5B**). Importantly, the highly skewed spectratype towards a particular CDR3 amino acid length was caused by a single Vγ gene in all animals and, to a lesser degree, in δ chains. The dominant Vγ clonotypes were found in all three animals on day 0, but this overlap diminished by day 21 (**Supplementary Figure 8**).

**Figure 6:**
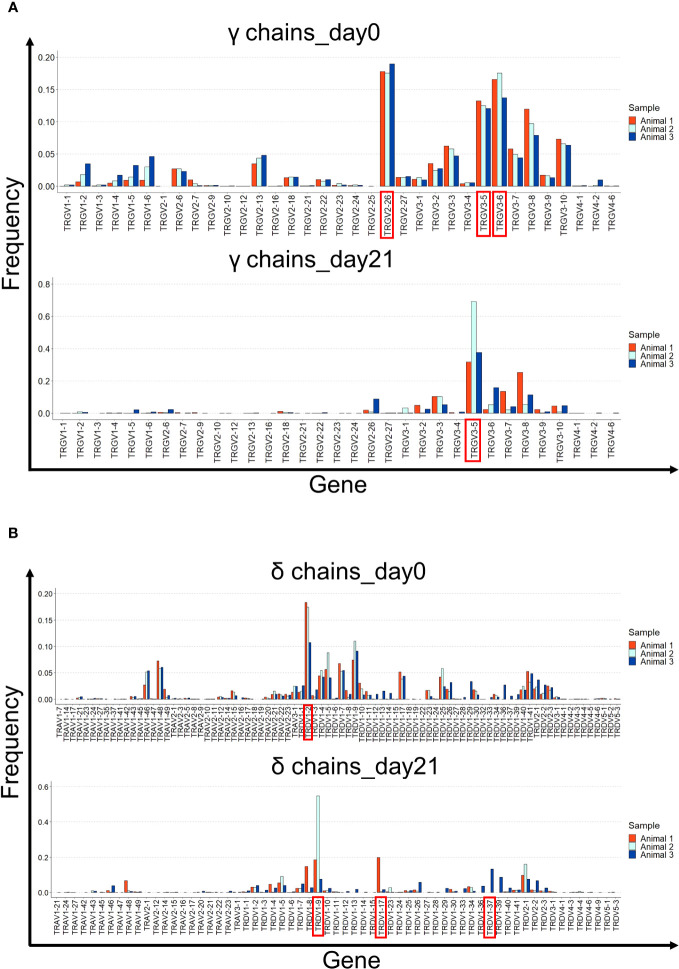
V gene usage in the γ and δ chain repertoires of cultured splenocytes. **(A)** Gene usage of the different Vγ segments is shown for the three animals on days 0 and 21 of cell culture. **(B)** Gene usage of the different Vδ segments is shown for the three animals on days 0 and 21 of cell culture. The most prevalent Vγ and Vδ segments are shown in red.

Next, we analyzed the gene usage of the Vγ and Vδ segments (**Figure 6**). On day 0, the dominant Vγ sequences were TRGV2-26, TRGV3-5, and TRGV3-6. However, by day 21, TRGV3-5 usage was predominant (**Figure 6A**). The gene usage pattern in the δ chains was different. TRDV1-2 was the predominant V gene by day 0, but not on day 21, where a different Vδ was overrepresented in every animal (TRDV1-17, TRDV1-9, and TRDV1-37) (**Figure 6B**).

On days 0 and 21, the most prevalent Jγ gene was TRGJ3, followed by TRGJ2 and TRGJ1. TRDJ1 was preferentially used in δ chains at both time points (**Supplementary Figure 9**).

Collectively, these data indicate that the γδ T cell repertoire is narrowed during culture with IL-2 and IL-12 because of the preferential expansion of T cells originating from the specific Vγ and Vδ genes. Notably, the predominant V genes and clonotypes on day 21 varied among the different animals.

## 4 Discussion

Chickens belong to γδ high T cell species; hence, a decent fraction of peripheral T cells in various organs bear the γδ TCR; however, the function of these cells is largely unknown. One reason for this lack of knowledge is the inability to culture these cells *ex vivo* under defined conditions. In this paper, we describe a system that can overcome this problem.

To establish selective outgrowth of chicken γδ T cells derived from total splenocyte preparations in cell culture, we used the cytokines IL-2 and IL-12. Our decision for the indicated dilutions was made following the titration of IL-2 and IL-12 alone or in combination. The highest proliferation indices were observed with a 1:800 dilution of IL-2 combined with a 1:80 dilution of IL-12. Consequently, this combination was used for cytokine stimulation of cells in all experiments. Freshly isolated and cryopreserved cells were used to determine the optimal culture conditions. The fact that both cell conditions yielded the same results confirmed the robustness of the system.

We have indicated cytokine dilutions in this manuscript because there are no adequate systems for quantifying chicken IL-2 and IL-12.

To repeat the stimulation protocol, other laboratories must establish proliferation systems to identify optimal cytokine concentrations. Commercially available chicken IL-2 and IL-12p40 exist, but we were not convinced of their effects in the different assays tested. The combination of IL-2 and IL-12 has already been successfully used in our laboratory to stimulate chicken γδ T cells in different experiments (40, 61, 62). Selective outgrowth of γδ T cells has also been demonstrated by different stimulation protocols in humans and chickens (37, 63, 64), and Ueta et al. demonstrated that IL-12 has a stimulating effect on γδ T cells but not on αβ T cells in humans (65). Moreover, Yang et al. observed that IL-12 leads to the expansion of a specific γδ T-cell subset (66). The combination of these two cytokines stimulates NK cells in humans and mice (67–70). IL-2 and IL-12 enhance the cytolytic effects of activated T cells in mammals (71), and it has been shown that a combination of IL-2 and IL-12 has a synergistic effect on chicken γδ T cells compared to IL-2 or IL-12 alone. IL-12 alone was not able to induce the cytolytic ability of γδ T cells, but the combination of IL-2 and IL-12 strongly induced cytotoxic effector functions (40). The results of BrdU proliferation assays (**Figure 1**) demonstrated that IL-12 alone barely promoted proliferation, whereas IL-2 boosted proliferation capacity. In previous experiments, we showed that IL-2 preferentially stimulated CD8^+^ cells in a 6-day culture system, whereas IL-18 induced outgrowth of CD4^+^ cells (48). However, the TCR phenotype of the cells was not analyzed further. The first attempts to cultivate γδ T cells were performed by Kasahara et al. (37), who demonstrated that proliferation was induced only by receptor ligation and cytokine-containing tissue culture supernatant. Moreover, stimulation was only observed in large CD8^+^ γδ T cells, as opposed to smaller CD8^−^ γδ T cells, as measured by forward scatter. Choi et al. (47) described a culture system for γδ T cells using IL-2 in combination with Concanavalin A (ConA) stimulation. Together, these studies argue in favor of a dual stimulation requirement, either by TCR crosslinking in combination with cytokine or dual cytokine stimulation, as demonstrated here. Lectin stimulation in splenocyte cultures most likely induces bystander cells to secrete IL-12. We propose that IL-2 or TCR crosslinking is important for upregulating the IL-12 receptor on γδ T cells; however, owing to the lack of reagents to detect the chicken IL-12 receptor, this could not be tested. Berndt et al. (72) introduced a PBL culture system using IL-2 in combination with PMA, which induced the proliferation of γδ T cells. We were not able to induce proliferation of blood γδ T cells or IEL by co-stimulation with IL-2 and IL-12. These differences may be explained by either the use of different chicken lines or PMA versus IL-12 stimulation used in these studies. As part of future studies, it would be interesting to investigate whether chicken γδ T cells in IL-2 and IL-12 culture systems produce cytokines, such as IFN-γ. This effector function has been described in other species, including pigs (73) and bovines (74), where γδ T cells stimulated with IL-2 and IL-12 (among other factors) produce IFN-γ. Different studies have also shown that chicken γδ T cells produce IFN-γ after infection with MDV (39, 75). It is particularly important to determine whether cytokine production and CD8 expression in chickens are related.

During the establishment of the *in vitro* culture system, we encountered several variables that influenced the outcome of the cultures. Splenocyte preparation on day 0 showed a range of 15%–60% TCR1^+^ cells. This is partially consistent with earlier studies, where a range of 20%–30% was observed (16, 37). The percentage of TCR1^+^ cells differed between female and male animals with 15% to 25% in females and 30 to 60% TCR1^+^ cells in males, respectively (**Supplementary Figure 10A**). An androgen-induced expansion of γδ T cells in the blood and spleen of male chickens of 4 months–6 months has been reported previously (38). The proliferation capacities of the male- and female-derived splenocytes were identical. The age of the donor chickens had an effect on culture outcomes. Splenocytes of animals older than 4 months started to proliferate 2 days–4 days earlier and more reliably than those of animals younger than 4 months (**Supplementary Figure 10B**).

In the flow cytometry measurements of splenocytes, we observed a distinct behavior of the different subpopulations over time: the double negative cells (TCR1^−^CD8^−^) were diminished, the TCR1^−^CD8^+^ cells decreased, and the TCR1^+^CD8^−^ cells remained in the same range, whereas the number of double-positive cells (TCR1^+^CD8^+^) increased (**Figure 2A**).

To further characterize the IL-2/IL-12 responsive cells (TCR1^+^CD8^+^ and TCR1^+^CD8^−^) in the splenocyte preparation, we performed sorting experiments of three populations based on γδ TCR and CD8 expression. With these experiments, we intended to address the following questions. First, is the proliferation dependent on bystander cells? Second, does only one of the phenotypes determine proliferation capacity? Third, do the different phenotypes remain stable in cell culture? We hypothesized that only CD8^+^ cells would proliferate. However, our experiments revealed that all of these populations showed vigorous proliferation with no difference between CD8^−^ and CD8^+^ cells in all animals tested, indicating that there is no need for other cells in culture that either secrete cytokines or are stimulated by cell–cell contact. In addition, the different TCR subsets proliferated, thus excluding the possibility of a subset that is solely reactive to cytokines. The phenotype, as judged by CD8 expression, was unstable during proliferation. On average, about one-third of TCR1^high^CD8^−^ cells upregulated CD8 after 7 days, and a fraction of the TCR1^low^ CD8^-^ cells expressed CD8 following stimulation, whereas the phenotype of TCR1^+^CD8^+^ cells remained stable. Thus, we conclude that stimulation leads to the expression of CD8 and that TCR density may be indicative of a previous activation. Interestingly, in experiments performed by Kasahara et al. (37) using negative-sort purified cells, TCR1^+^CD8^+^ cells responded well in the presence of ConA or anti-CD3 in combination with exogenous growth factors, whereas CD8^−^ cells did not. The differences in CD8^−^ cell proliferation observed in our study may be the result of different stimulation protocols. Studies on *Salmonella typhimurium* by Berndt et al. (42, 43), for example, showed an increase in CD8^+^TCR1^+^ cells after infection. These findings are consistent with our results, but we cannot conclude that there was a change in the CD8 phenotype of the expanding cells, as we lacked information regarding their initial CD8 status.

In the next step of our analyses, we took advantage of a recently developed protocol for TCR profiling in our laboratory (Früh et al. in preparation). This is based on 5’ RACE with a primer specific to the constant C region and a template switch oligo, including a unique molecular identifier at the 5’ end. TCR variable region amplicons were then generated by two successive rounds of semi-nested PCRs performed on the cDNA, followed by Illumina sequencing. These experiments on mRNA derived from days 0 to 21 of culture were performed to analyze whether only specific clonotypes for gamma and delta were preferentially stimulated by IL-2 and IL-12 among the examined animals and would therefore be responsible for the long-living cells in the culture, or alternatively, whether dominant clonotypes were an individual outcome after stimulation. For example, in humans, IL-12 causes expansion and differentiation of a specific γδ T cell subpopulation, namely Vγ2Vδ2 T cells (66).

Changes in the γ and δ repertoires were recognized in different aspects. Long-term culture led to a smaller number of expressed V regions for gamma and delta chains; the expressed V regions appeared at a higher frequency on day 21, and the dominant clonotypes differed between days 0 and 21. Cell proliferation appears to be independent of the frequency of clonotypes present on day 0, as the most prevalent clonotypes on day 21 do not align with those observed as the most prevalent on day 0 and vary between animals. Thus, the individual outcomes of different cultures may be the result of previous *in vivo* activation and *in vitro* expansion of cells. This was less pronounced in animal 3, in which the repertoire after stimulation was less restricted. Interestingly, this animal showed the highest proliferation capacity after three weeks of culture. Notably, three weeks after stimulation, the most frequently used Vγ gene family was identical in all animals.

So far, only a few analyses of the TCR repertoire have been conducted in chickens. In a study by Dixon et al., a single TRGV gene, TRGV3.3, was dominant in all tissues analyzed. This gene comprises 30%–40% of the entire TCR gamma repertoire (8). In contrast, we were unable to identify a single dominant TRGV gene on day 0. This difference may be due to the different chicken genome sequences and lines used for the analyses.

In conclusion, our data demonstrated that chicken γδ T cells can be stimulated for extended periods with IL-2 and IL-12 in cell culture. In this culture, there is a shift to a more restricted repertoire. This culture system will be very useful for characterizing γδ T cell function in future experiments and for obtaining more information regarding γδ TCR ligands.

## Data availability statement

The data presented in the study are deposited in the SRA database, accession number PRJNA1054968.

## Ethics statement

The animal study was approved by Government of Upper Bavaria, identification code: 55.2-1-54-2532.0-60-2015; June, 2019. The study was conducted in accordance with the local legislation and institutional requirements.

## Author contributions

AL: Investigation, Writing – original draft, Conceptualization, Formal analysis. SF: Writing – review & editing, Conceptualization, Formal analysis. TG: Writing – review & editing, Conceptualization, Formal analysis, Funding acquisition.
