# Supplementary material for: Chicken γδ T cells proliferate upon IL-2 and IL-12 treatment and show a restricted receptor repertoire in cell culture
Source: Front Immunol. 2024 Feb 13;15:1325024. doi: 10.3389/fimmu.2024.1325024 (PMC10900522; doi:10.3389/fimmu.2024.1325024)
Supplement: Supplementary file 1 [file DataSheet_1.docx]

Supplementary Material

Chicken γδ T cells proliferate upon IL-2 and IL-12 treatment and show a restricted receptor repertoire in cell culture

Antonia E. Linti, Thomas W. Göbel^*^, Simon P. Früh

*** Correspondence:** Thomas W. Göbel: [goebel@lmu.de](mailto:goebel@lmu.de)

**Suppl. Fig. 1**

**
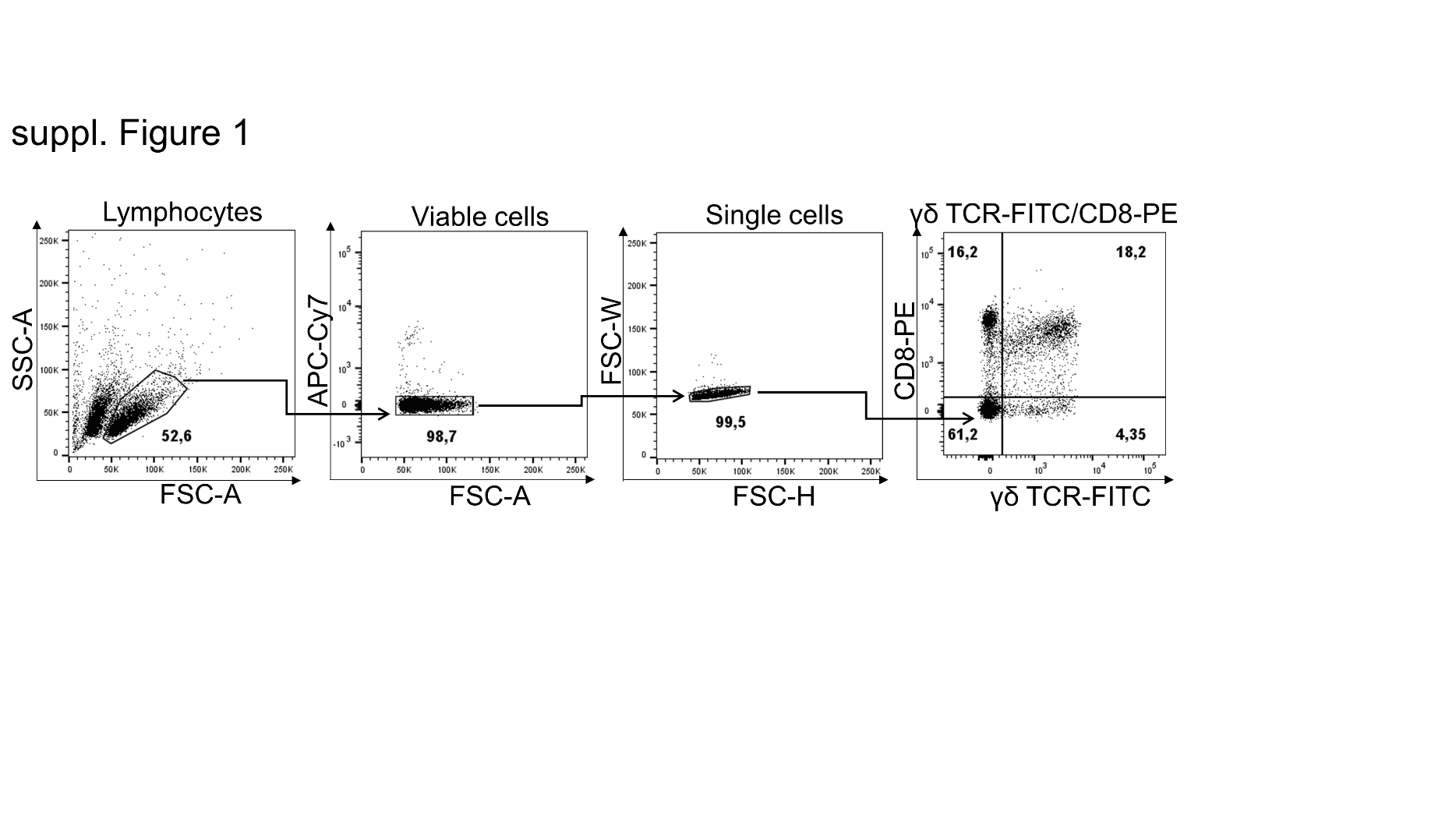
**

**Suppl. Fig. 1:** Gating strategy for flow cytometry analysis.

Splenocytes were isolated by density-gradient centrifugation and stained with TCR1 and CT8 mAbs, and a Fixable Viability Dye. Percentages of the different cell populations are indicated.

**Suppl. Fig. 2**

**
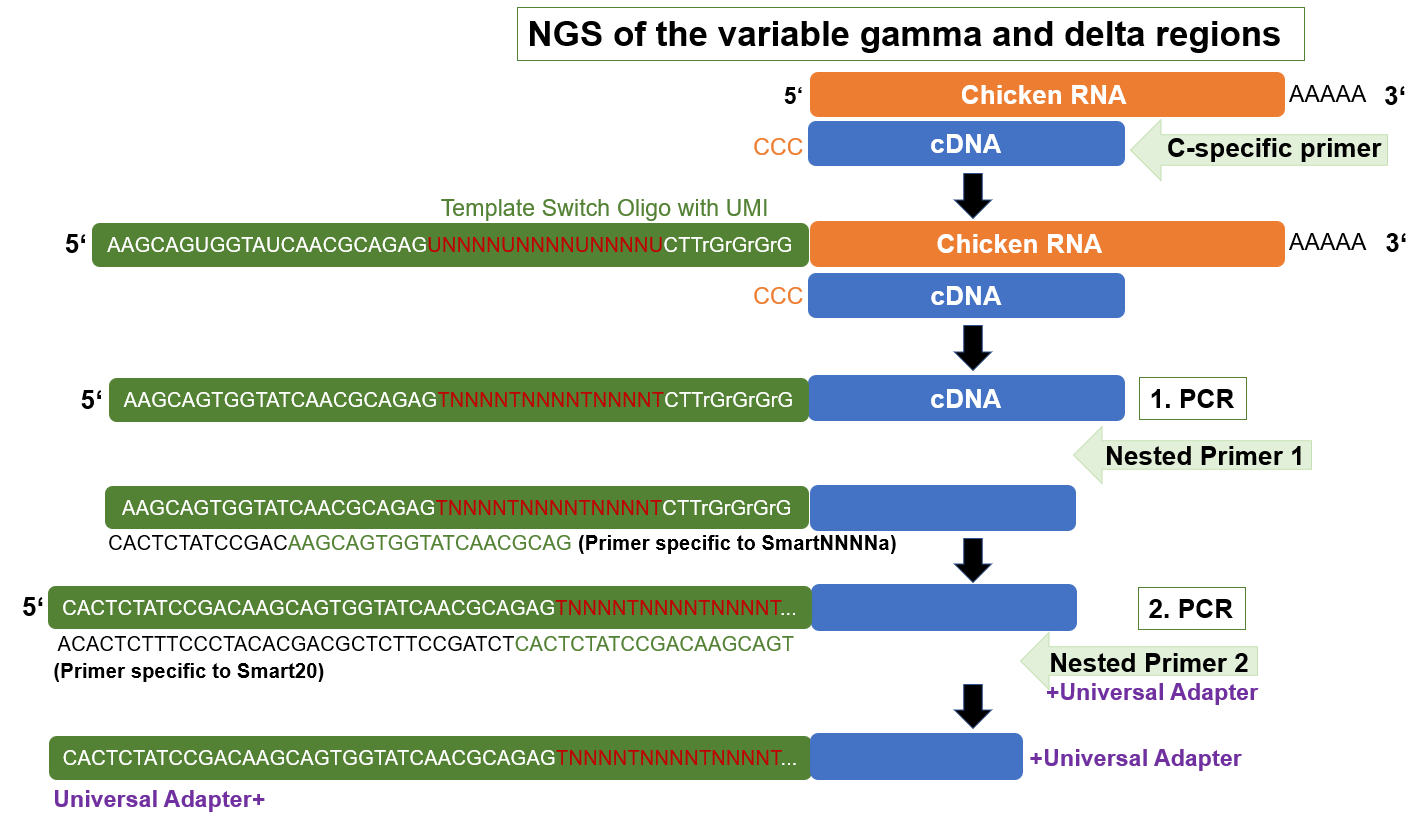
**

**Suppl. Fig. 2:** Amplification of TCR γδ variable regions.

Schematic overview of cDNA synthesis and semi-nested PCR amplification of TCR variable regions with Primers used in each step.

**Suppl. Fig. 3**

(A) (B) (C)

**
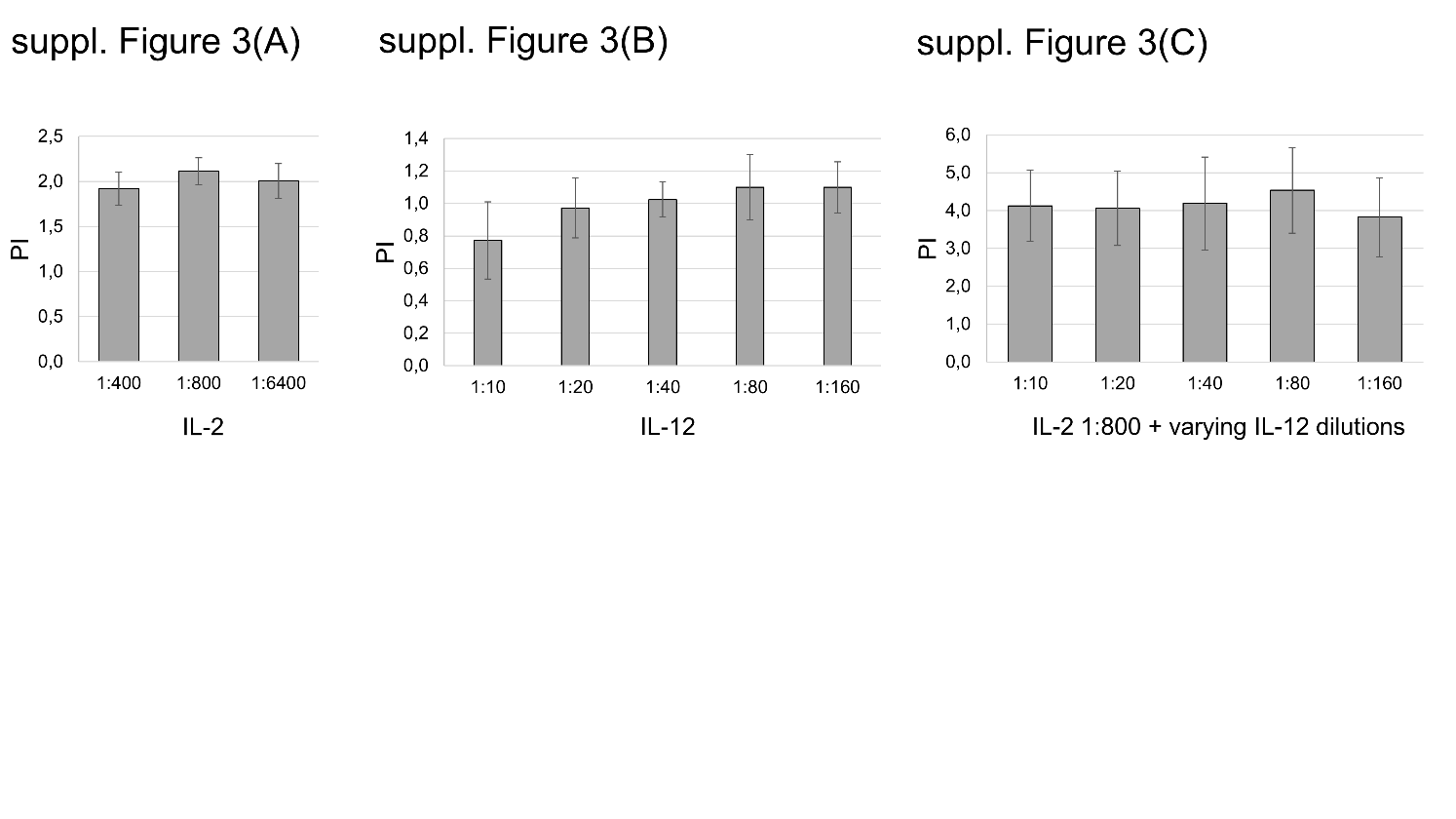
**

**Suppl. Fig. 3:** Titrations of IL-2 and IL-12.

(A) BrdU assay of splenocytes stimulated with IL-2 at various dilutions (initial dilution 1:400, 2-fold dilutions). (B) BrdU assay of splenocytes stimulated with IL-12 at various dilutions (initial dilution 1:10, 2-fold dilutions). (C) BrdU assay of splenocytes stimulated with a combination of IL-2 (1:800) and IL-12 at various dilutions (initial dilution 1:10, 2-fold dilutions). For (A), (B) and (C), animals = 3. Mean ± SD; p-values in (A), (B) and (C) p > 0.05 = not significant.

**Suppl. Fig. 4**

(A) (B)

**
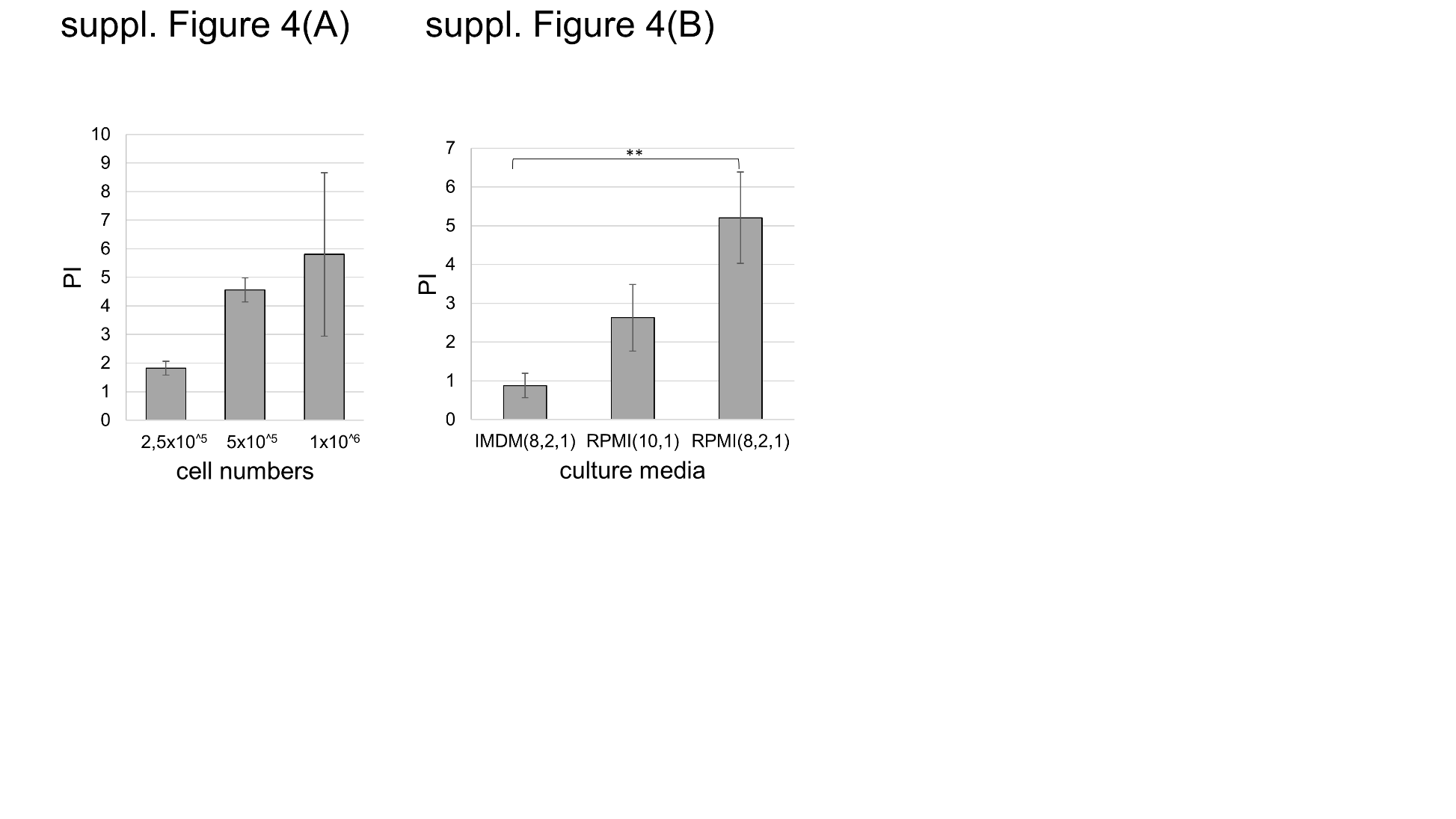
**

**Suppl. Fig. 4:** Comparison of proliferation with varying seeding densities and different cell culture media.

(A) BrdU assay of splenocytes stimulated with IL-2 (1:800) and IL-12 (1:80). Proliferation indices of splenocytes at different seeding densities (2,5x10^5, 5x10^5 and 1x10^6 cells/well). (B) BrdU assay of splenocytes stimulated with IL-2 (1:800) and IL-12 (1:80). Proliferation indices of cells in different culture media (IMDM 8%FBS, 2%ChS and 1%P/S, RPMI 10%FBS and 1%P/S and RPMI 8%FBS, 2%ChS and 1%P/S). For (A) and (B), animals = 3. Mean ± SD; p-values in (A) and (B) p > 0.05 = not significant, unless indicated otherwise: ** = p ≤ 0.01.

**Suppl. Fig. 5**

**
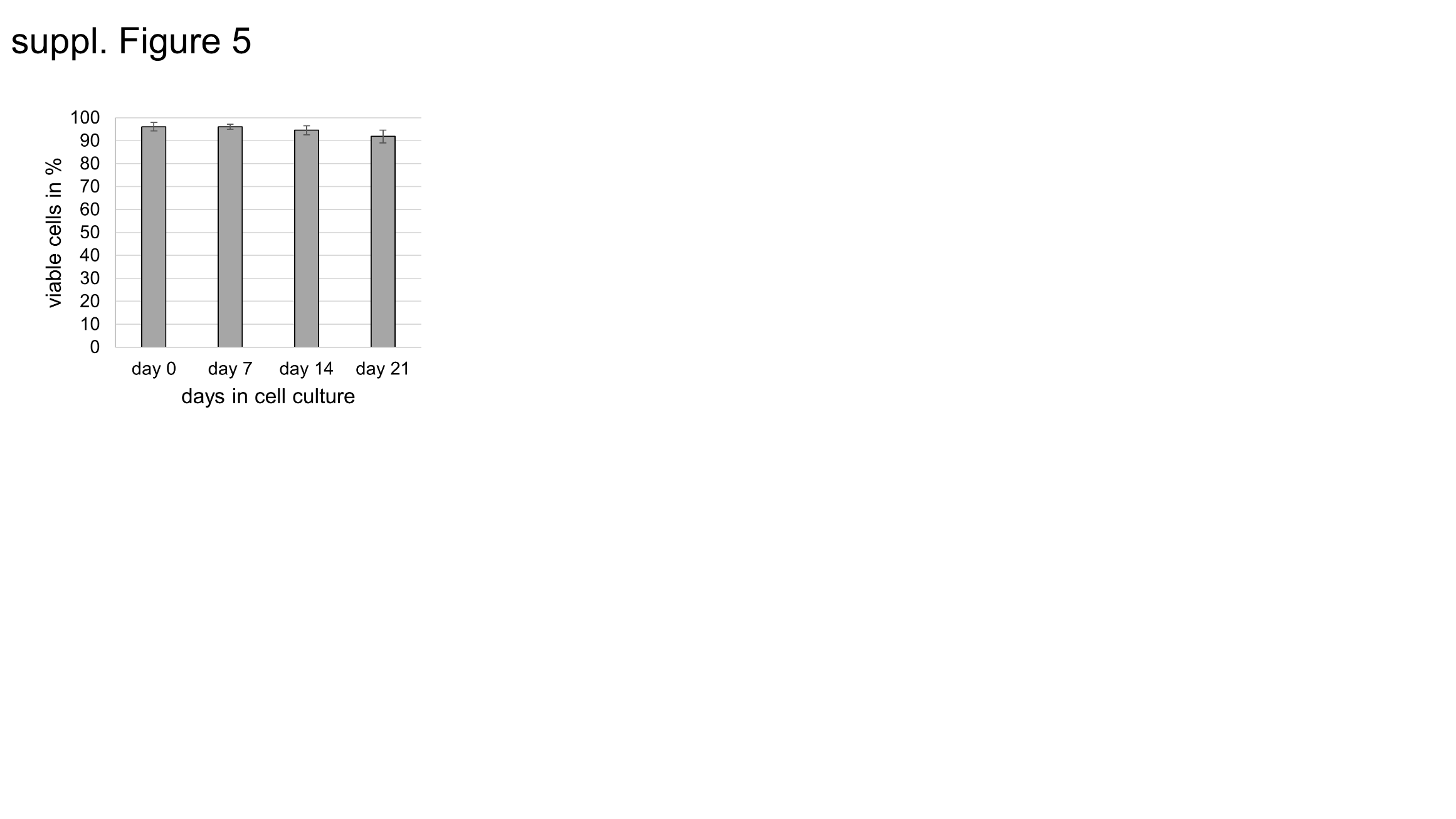
**

**Suppl. Fig. 5:** Cell viability of splenocytes stimulated with IL-2 and IL-12 over time.

Cell viability (% of lymphocytes) of splenocytes stimulated with IL-2 and IL-12 was determined by staining with a Fixable Viability Dye and flow cytometry analysis on day 0, day 7, day 14 and day 21. Animals = 3; Mean ± SD.

**Suppl. Fig. 6**

**
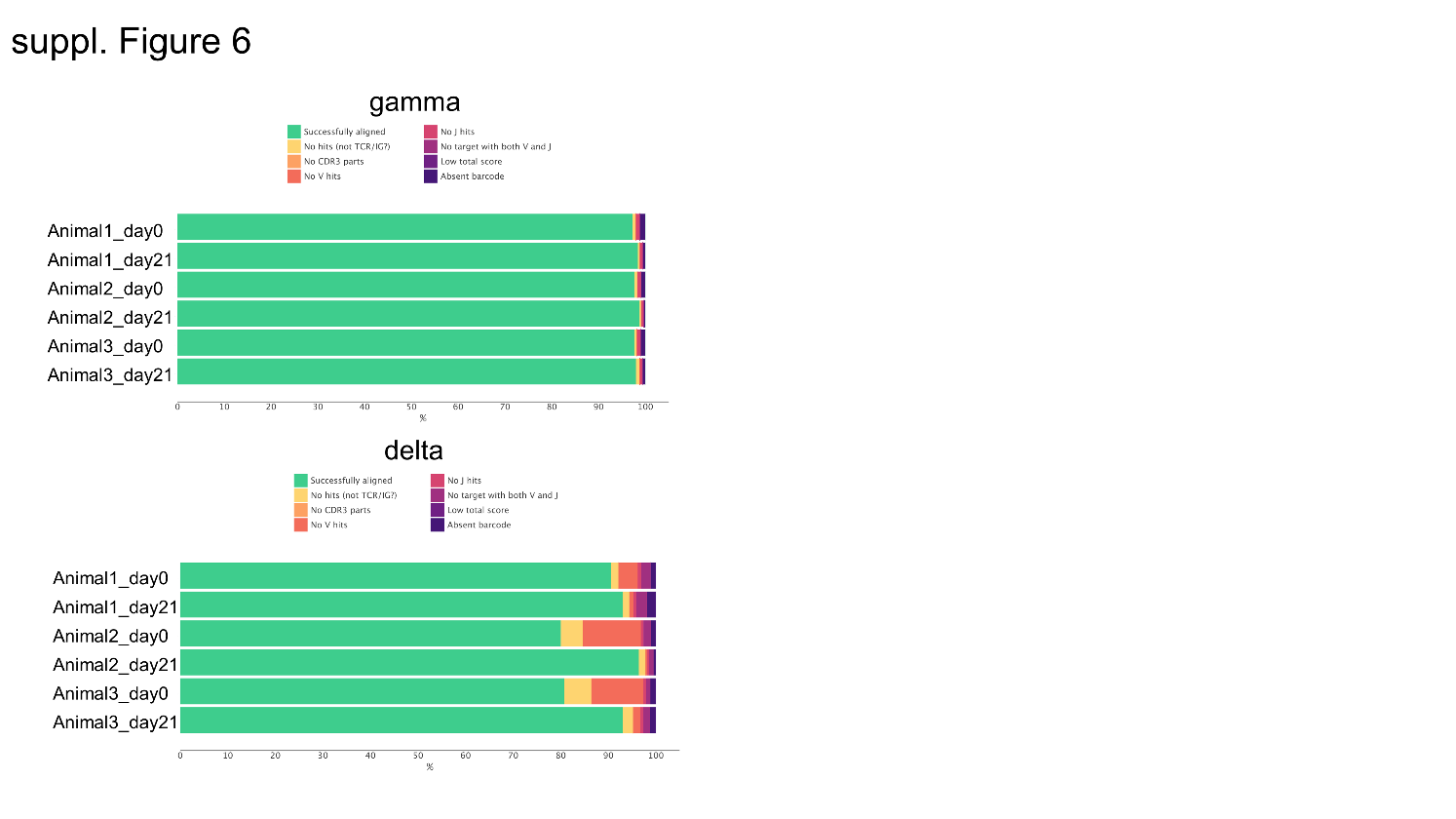
**

**Suppl. Fig. 6**: Alignment rate of raw sequences using MiXCR and a custom V(D)J reference gene library.

Successfully aligned sequences (green) are shown for γ (top) and δ (bottom) chain amplicons from three animals at day 0 and day 21.

**Suppl. Fig. 7**

**
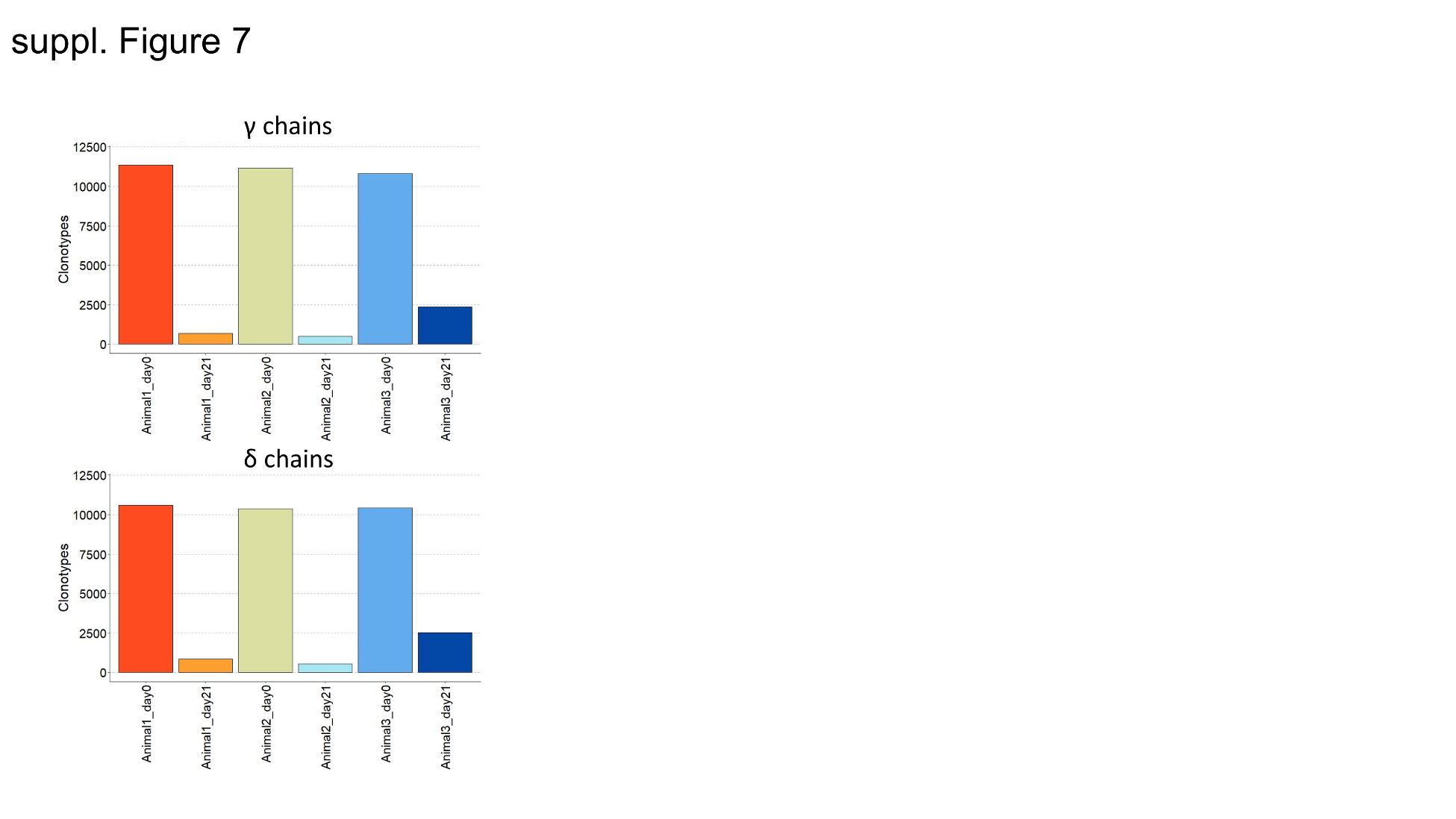
**

**Suppl. Fig. 7:** Number of unique clonotypes in γ and δ chain repertoires on day 0 and day 21 of cell culture.

For each animal the number of unique clonotypes on day 0 and day 21 (Animal1_day0, Animal1_day21, Animal2_day0, Animal2_day21, Animal3_day0, Animal3_day21) is indicated for γ and δ chains.

**Suppl. Fig. 8**

**
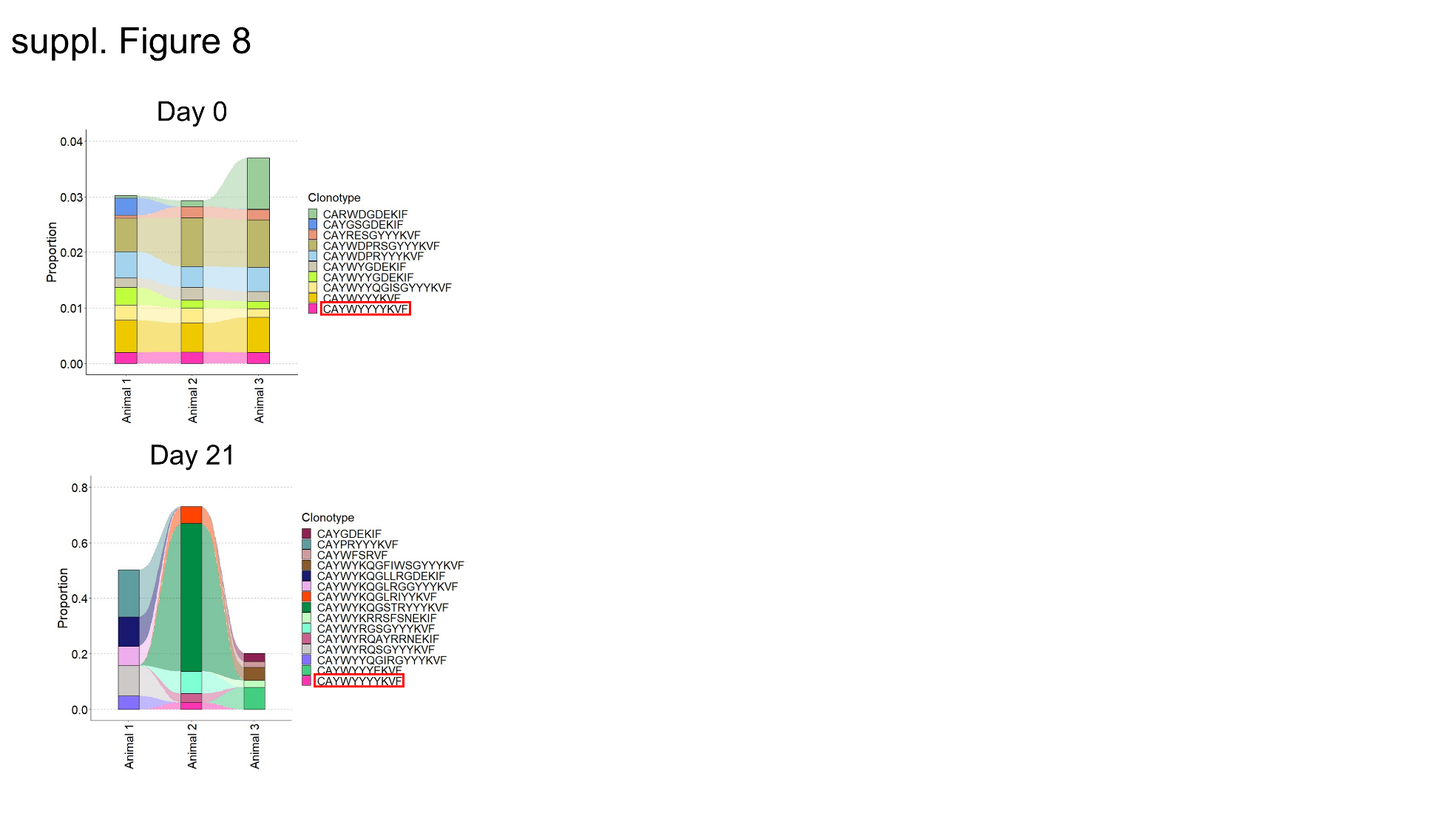
**

**Suppl. Fig. 8:** Clonotype tracking of the most abundant clonotypes on day 0 and day 21 for γ chain TCR sequences.

The most abundant γ chain CDR3 clonotypes (see legend) across animals were compared on day 0 and day 21. Red box indicates a clonotype that was among the most frequent clonotypes on day 0 and day 21 of cell culture. Animals = 3.

**Suppl. Fig. 9**

**
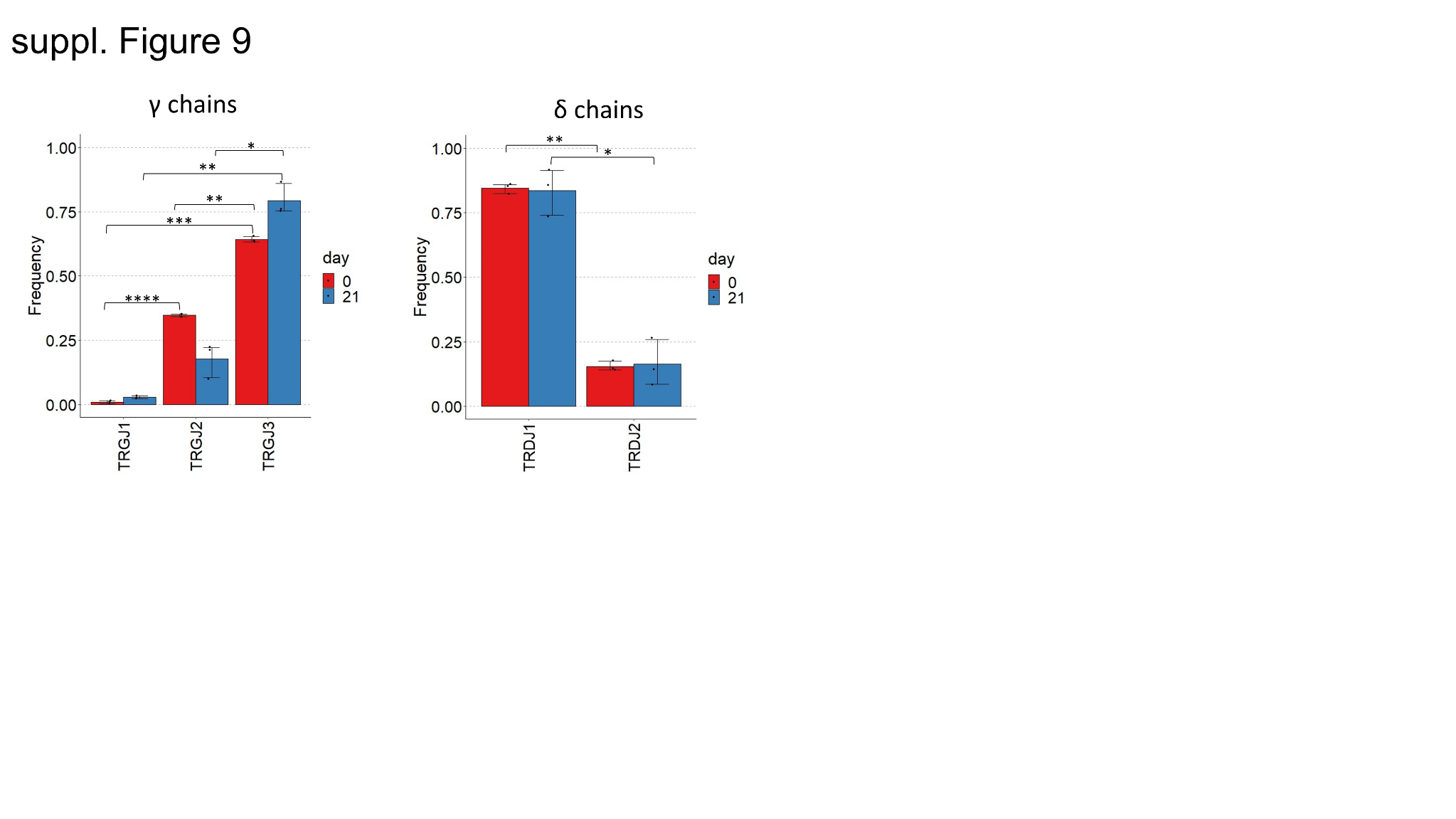
**

**Suppl. Fig. 9:** Jγ and Jδ gene usage on day 0 and day 21.

J gene usage of the different Jγ and Jδ segments is shown for three animals on day 0 and day 21 (see legend). p-values as indicated, * = p ≤ 0.05, ** = p ≤ 0.01, *** = p ≤ 0.001, **** = p ≤ 0.0001. All other comparisons: p > 0.05 = not significant.

**Suppl. Fig. 10**

(A) (B)

**
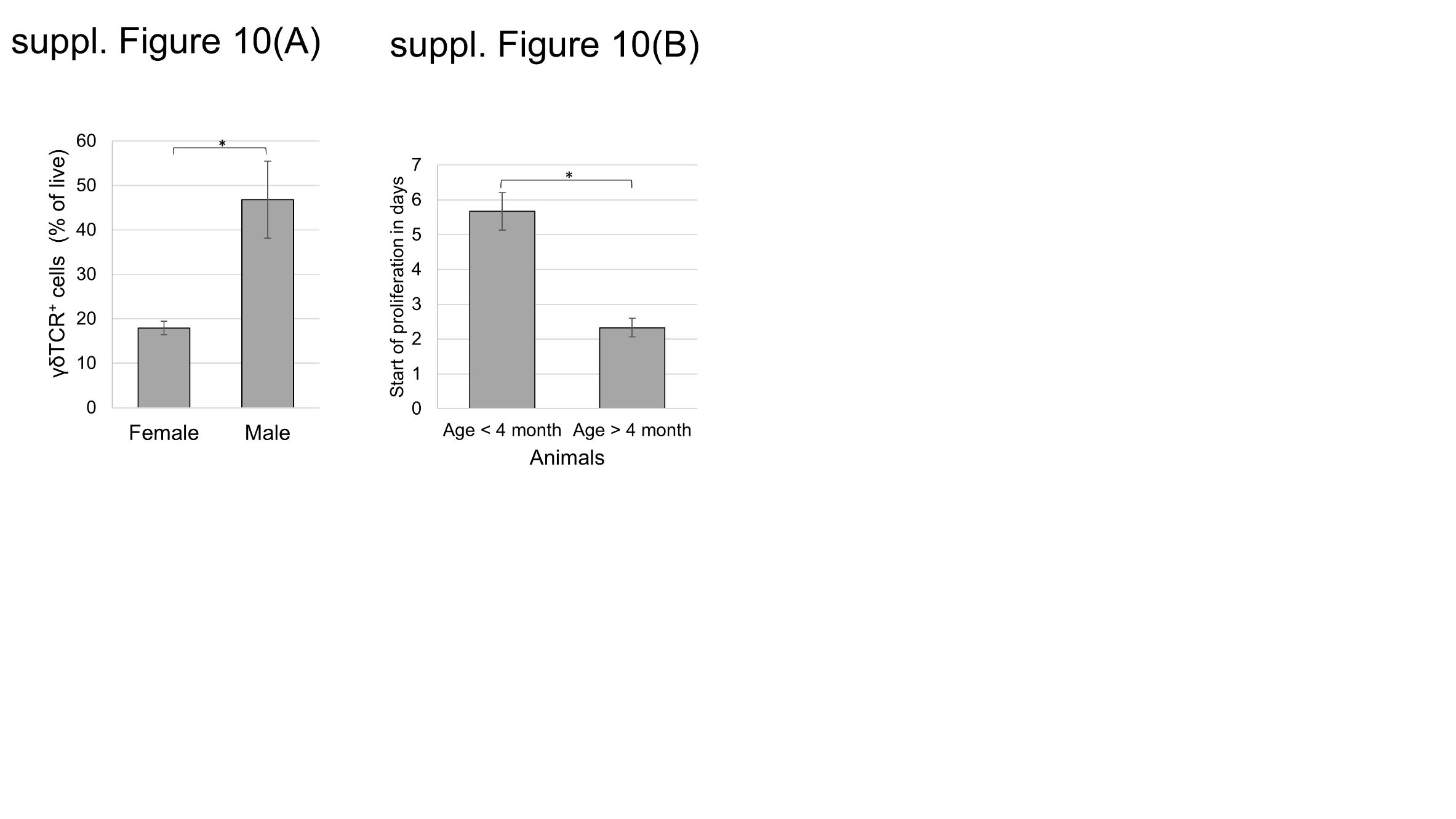
**

**Suppl. Fig. 10:** Impact of sex and age on γδ T cell frequency and proliferation.

(A) γδ TCR frequencies in freshly isolated splenocytes on day 0 depicted separately for female and male animals (each = 3) (% of live lymphocytes). (B) The start of proliferation comparing animals younger than 4 months with animals older than 4 months (each = 3). (A) and (B): Mean ± SD; p-values as indicated, * = p ≤ 0.05.

**Suppl. Table 1**: cDNA-synthesis for gamma and delta.

| **Primer Annealing** | 72°C for 3min  42°C for 2min | - Mix1 (4,5µl): specific primers (0,5µM each) and RNA |
| --- | --- | --- |
| **Reverse Transcription** | 42°C for 90min  70°C for 10min | - Mix2 (5,5µl): reverse transcriptase (10U), buffer (1x), dNTPs (1mM each), DTT (2,5mM), oligonucleotide with UMI (1µM), RNase inhibitor (1U) - Mix2 to Mix1 (10µl) - Ligation of 5’ oligonucleotide |
| **Digest of unbound UMI** | 37°C for 60min | - Add 5µl USER® Enzyme per reaction (Uracil DNA glycosylase, NEB) |
| **Storage** | -20°C | - Storage temperature |

**Suppl. Table 2**: List of used primers for cDNA synthesis, first and second PCR.

|  | Oligonucleotide with integrated UMI | forward | SmartNNNNa | AAGCAGUGGTAUCAACGCAGAGUNNNNUNNNNUNNNNUCTTrGrGrGrG |
| --- | --- | --- | --- | --- |
| **cDNA synthesis** | Specific primer to C region gamma | reverse | CHTCRg_1 | CATCGGTCCATTTCACCCGA |
|  | Specific primer to C region delta | reverse | CHTCRd_1 | TCATTAGAGGACATCTCCAAA |
|  | Primer specific to SmartNNNNa | forward | Smart20 | CACTCTATCCGACAAGCAGTGGTATCAACGCAG |
| **First PCR** | Nested primer C-region gamma | reverse | CHTCRg_2 | TCATGTTCCTCCTGCATGATTTC |
|  | Nested primer C-region delta | reverse | CHTCRd_2 | TGATTTCATCACAATGACCTCTGG |
|  | Primer specific to Smart20 with Universal Adapter gamma | forward | Step_1_g | (ACACTCTTTCCCTACACGACGCTCTTCCGATCT)CACTCTATCCGACAAGCAGT |
| **Second PCR** | Primer specific to Smart20 with Universal Adapter delta and a barcode | forward | Step_1_d | (ACACTCTTTCCCTACACGACGCTCTTCCGATCT)CGCAACACTCTATCCGACAAGCAGT |
|  | Nested primer C-region with Universal Adapter gamma | reverse | CHTCRg_3 | (GACTGGAGTTCAGACGTGTGCTCTTCCGATCT)CTGGTGCTGAACTTCCTTTGTC |
|  | Nested primer C-region with Universal Adapter delta and a barcode | reverse | CHTCRd_3 | (GACTGGAGTTCAGACGTGTGCTCTTCCGATCT)GCTGCGAATAGAATCTCTCTGCTCCC |

**Suppl. Table 3**: PCR cycling conditions for the first and second PCR.

|  | **First PCR** | **Second PCR** |
| --- | --- | --- |
| **Initial Denaturation** | 95°C for 1min | 95°C for 1min |
| **Denaturation**  **Annealing**  **Elongation** | 95°C for 20sec  65°C for 20sec  72°C for 50sec | 95°C for 20sec  65°C for 20sec  72°C for 50sec |
| **Final Elongation** | 72°C for 2min | 72°C for 5min |
| **Number of cycles** | For gamma + delta: 18 | For gamma 12, for delta 15 |
